# Potatoes – a scoping review for Nordic Nutrition Recommendations 2023

**DOI:** 10.29219/fnr.v68.10454

**Published:** 2024-01-29

**Authors:** Magdalena Rosell, Christine Delisle Nyström

**Affiliations:** Department of Biosciences and Nutrition, Karolinska Institutet, Stockholm, Sweden

**Keywords:** potatoes, solanum tuberosum, starchy foods, dietary guidelines

## Abstract

Potatoes comprise a common staple food in the Nordic and Baltic countries and contribute to the diet with vitamins, minerals, dietary fibre and phytochemicals. However, potatoes may also be consumed in processed forms with added fat and salt, which raises concerns about possible adverse health effects. The aim of this scoping review is to describe the overall evidence for the role of potatoes as a basis for setting and updating food-based dietary guidelines in the Nordic Nutrition Recommendations 2023. PubMed was searched for systematic reviews and meta-analyses, and evidence was extracted on relevant health outcomes. Current available evidence indicates that moderate consumption of potatoes is not associated with a substantial risk of chronic diseases, and that they may be part of a healthy diet. However, the health effects vary greatly depending on cooking methods, and studies indicate that the intake of French fries/fried potatoes should be limited. Overall, the evidence regarding health effects of potatoes is very limited, and possible associations need to be further investigated.

## Popular scientific summary

- Potatoes are a staple food in the Nordic and Baltic countries.
- Potatoes are a source of several micronutrients like vitamin C and potassium as well as dietary fibre, high-quality protein and phytochemicals.
- While potatoes are rich in starch and can have a high glycaemic index, the glycaemic load is often lower compared with other starchy foods.
- Recent evidence indicates that moderate consumption of potatoes may be part of a healthy diet and is not associated with a substantial risk of chronic diseases.
- Studies indicate that the intake of French fries/fried potatoes should be limited.

Potatoes (*Solanum tuberosum*) comprise a commonly consumed staple food in the Western world and are available all year around due to their ability to withstand long storage (1). Because of their high content of starch, potatoes are often separated from other vegetables and not included in the recommended daily intake of fruits and vegetables. However, like other vegetables, they have a high content of water and contain less energy and carbohydrates compared to other common starchy foods, such as cooked pasta or brown rice (1). They are also significant contributors of nutrients, including vitamin C, vitamin B6, niacin, folate, potassium, calcium, phosphorous, iron and magnesium, and they also contain dietary fibre (mainly in the peel), protein of high quality and phytochemicals, such as phenolics and carotenoids (1).

The nutritional content may vary widely depending on the variety, growing environment, storage and cooking method (1–3). For example, boiling potatoes might reduce the content of vitamins, minerals and water-soluble phenols because of the leaching effect, but cooking may also facilitate the digestion of the protein, increase the amount of compounds with antioxidative properties and increase the content of fibre due to formation of complexes between polysaccharides and other compounds and the formation of resistant starch (1, 3). The glycaemic index (GI) also varies depending on the variety, cooking method and whether the potato is served hot or cold. Generally, a higher GI is observed for mashed and boiled potatoes compared with fried, microwaved or baked potatoes (3, 4). Storing cooked potatoes at a lower temperature prior to consumption will also increase the content of resistant starch and decrease the GI (3). The content of fat is naturally low in potatoes, but it may increase considerably by adding fat through frying or roasting them with oil or by adding fat containing sauce or gravy, which also raises concern regarding possible adverse health effects (1). Potatoes may also be consumed in the form of crisps and other types of potato snacks; these are not further discussed in this review.

There are many factors affecting the nutritional value making it complex to study the health effects of potatoes. This scoping review will summarise the evidence for the role of potatoes for health-related outcomes as a basis for setting and updating food-based dietary guidelines in the Nordic Nutrition Recommendations 2023 (Box 1). The search strategy was focused on systematic reviews and meta-analyses of observational and intervention studies summarising epidemiologic evidence in this area.

Box 1 Background papers for Nordic Nutrition Recommendations 2023

- This paper is one of many scoping reviews commissioned as part of the Nordic Nutrition Recommendations 2023 (NNR2023) project (4)
- The papers are included in the extended NNR2023 report but, for transparency, these scoping reviews are also published in Food & Nutrition Research
- The scoping reviews have been peer reviewed by independent experts in the research field according to the standard procedures of the journal
- The scoping reviews have also been subjected to public consultations (see report to be published by the NNR2023 project)
- The NNR2023 committee has served as the editorial board
- While these papers are a main fundament, the NNR2023 committee has the sole responsibility for setting dietary reference values in the NNR2023 project

## Methods

This scoping review is based on the same methodology and search strategy described previously (5). Systematic reviews and meta-analyses for potatoes were selected in the same literature search. One qualified systematic review (qSR) was identified regarding vegetables, fruits and berries, namely the Continuous Update Project Expert Report 2018 from the World Cancer Research Fund/American Institute of Cancer Research (WCRF/AICR) on wholegrains, vegetables and fruit and the risk of cancer (6). However, this report did not present evidence regarding potatoes. One qSR was used for the current review (7). Of the 166 articles identified in the main search, nine articles investigated potatoes. Two articles included cancer as an outcome, but as cancer was not specifically searched for in the main search, an additional search was done using the following search string:

potato*[Title/Abstract] AND “cancer[Title/Abstract] AND 2011:2022[pdat] AND (meta-analysis[Filter] OR systematicreview[Filter]).

This search generated 12 articles, of which one additional article was identified regarding cancer, resulting in 10 articles in total. For the summary below, six articles were selected based on the most recent reviews, comprehensiveness and quality checked by using the modified AMSTAR 2-NNR (Supplementary material) (8, 9). Systematic reviews and meta-analyses of prospective studies and randomised controlled trials were of primary interest. For the section on mechanisms, a general search approach was also used.

## Dietary intake in Nordic and Baltic countries

According to data from national dietary surveys in adults, the mean intake of potatoes ranges between around 50 and 130 g/day among the different Nordic and Baltic countries, with large individual variations within the countries (10). The highest intakes are seen in Sweden, Denmark, Estonia and Latvia, and in all countries, higher intakes are seen in men than in women (10).

## Health outcomes relevant for Nordic and Baltic countries

In one qSR from 2013 (7), no conclusion could be drawn regarding the associations between potato consumption and cardiovascular disease/coronary heart disease (CVD/CHD), type 2 diabetes or inflammatory markers. Recent meta-analyses of prospective studies have reported no associations between total intake of potatoes and cardiovascular disease and all-cause mortality (11, 12). Studies were too few to analyse the potatoes prepared with different cooking methods (11, 12). For overall cancer, no association was seen when comparing high versus low intake, but a positive non-linear association was indicated (12). An increased risk was seen specifically for total colorectal cancer when comparing high versus low intake, particularly seen in studies conducted in Europe (13). No differences between potatoes prepared with different cooking methods (fried vs. boiled/mashed/roasted) were demonstrated (13). In all three studies, the quality of the evidence was graded as low (11–13).

Regarding type 2 diabetes, the most recent meta-analysis showed no association for total potatoes comparing high versus low intake, while a positive dose–response association was observed for total potato intake, but not for boiled potatoes (14). No significant heterogeneity between subgroups was seen. The evidence was considered limited suggestive for total potatoes and limited-no conclusion for boiled potatoes, using the WCRF criteria (14). Another recent meta-analysis also showed an increased risk for total potato consumption (15), and in subgroup analyses the association was significant only for French fries and not for boiled potatoes (15). Indications of an association between total potato intake and risk for gestational diabetes has also been reported (16); no analyses were done regarding the cooking method.

In a systematic review of prospective studies, an association between the intake of potatoes and an increased risk of body weight was suggested, and a stronger association for French fries was indicated (17). However, the evidence was not conclusive and considered uncertain (17). An association between the intake of French fries and an increased risk of hypertension has also been reported in a dose–response analysis, while this was not seen for boiled/baked/mashed potatoes (12). The quality of evidence was considered moderate (12).

## Mechanisms

Potatoes may play a beneficial role in the diet because of their content of vitamins and minerals such as vitamin C, vitamin B6, calcium, iron and magnesium and also phytochemicals and dietary fibre. They are particularly high in potassium, which may be beneficial for, for example, preventing hypertension and bone loss (18). However, they are also rich in starch and have been considered high GI-foods (4), leading to a rapid increase in blood glucose and insulin, which could mediate adverse effects. However, the GI of potatoes may vary depending on the variety, cooking methods and whether the potato is served hot or cold (3, 4), which needs to be considered when analysing these associations. Also, the glycaemic load (GL), which takes the amount of carbohydrates typically consumed into consideration, is often lower for potatoes compared with, for example, pasta and white rice (1, 4). Consuming potatoes together with other foods may also reduce the GI considerably (2). Possible adverse effects of potato consumption could also be related to weight gain (2). However, studies have also indicated that isoenergetic portions of potatoes, particularly boiled potatoes, generates a higher satiation compared with other starchy carbohydrates when consumed in isolation (2), which may not support an association between potatoes and an increased risk of excess energy intake and weight gain. The evidence is still very limited though (2). Regarding fried potatoes, and particularly deep-fried potatoes, the content of fat and energy is considerably higher compared with, for example, boiled or baked potatoes, which could lead to an excessive energy intake, despite a relative lower GI compared with other cooked variants of potatoes. The fat content of pre-fried frozen potatoes heated in the oven in the household can vary and is generally lower than in deep-fried potatoes (19). The addition of salt may also have adverse effects on the cardiovascular system (20).

## Food-based dietary guidelines

Current available evidence indicates that potato consumption is not associated with cardiovascular disease and all-cause mortality, while the association between a high intake of potatoes and certain cancer types remains unclear. Adverse associations have also been reported for type 2 diabetes, particularly for French fries/fried potatoes. In one qSRs (7), no conclusion could be drawn regarding the associations between potato consumption and CVD/CHD, type 2 diabetes or inflammatory markers. Overall, the evidence is very limited and mainly based on observational prospective studies.

Potatoes comprise a common staple food in the Nordic and Baltic countries, and they contribute to the diet with vitamins, minerals, dietary fibre and phytochemicals and may in this respect be part of a healthy diet. However, possible health effects of potatoes may vary greatly depending on the cooking methods, and studies suggest that the intake of French fries/fried potatoes should be limited. This is further supported by a qSR on dietary patterns (21), which identified French fries/fried potatoes as a component of a dietary pattern associated with an increased risk of overweight and obesity in children, although the evidence was graded as limited. The same report also indicated that French fries/fried potatoes, as well as total potatoes as components of a dietary pattern were associated with an increased risk of colorectal cancer in adults; the evidence was graded as moderate (21). Potatoes were not identified as a consistent component of a dietary pattern associated with type 2 diabetes (21). Overall, this highlights the need for further research regarding the intake of potatoes and health.

The knowledge about possible health effects of potatoes is still limited, and areas for future research includes further investigations of potatoes using different study designs and taking different varieties of potatoes and different cooking methods into consideration. It may also be relevant to study possible health effects of potatoes in different geographical regions and further investigate the role of potatoes in different cultural contexts.

The point of departure of this scoping review is the concurrent scoping review for vegetables, fruits and berries and has similar limitations described previously (5). The search strategy was focused on systematic reviews and meta-analyses; only and newly published original studies on possible health effects of potatoes could have added further information.
